# Policing and public health calls for service in Philadelphia

**DOI:** 10.1186/s40163-021-00141-0

**Published:** 2021-03-02

**Authors:** Jerry H. Ratcliffe

**Affiliations:** Department of Criminal Justice, Temple University, Philadelphia, PA USA

**Keywords:** Philadelphia, Police, Public health, Medical, Calls for service, CAD

## Abstract

This contribution outlines various spatial and temporal aspects of medical or public-health related calls for service from the public to police in Philadelphia in 2019. These incidents comprise about 8% of the police department’s workload that originates from the public. Calls appear to be highly concentrated in a few areas, and specifically the Center City and Kensington neighborhoods. They are also more likely to occur late afternoon and evening. The article shows that some medical or public health activity initially masquerades as crime or other policing work and some events eventually determined to be police/crime activity can initially appear to be public health related. About 20% of activity in this area does not appear predictable from the initial call type as handled by police dispatch.

## Introduction

Researchers have long recognized that the police role stretches beyond crime enforcement to social order and peacekeeping (Bittner, 1967; Goldstein, 1979). Public health issues are also ‘inseparable’ from policing (Wood, 2020). Police regularly encounter vulnerable groups, including people struggling with mental illness, domestic and sexual violence, challenges related to sex work and drug addiction, alcohol abuse, and human trafficking (Dijk & Crofts 2017). These conditions can present together in multiple ways that challenge police officers to address the complexity of co-morbidity issues, often within a ‘vacuum’ of support from other organizations (Wood et al., 2021). Given numerous cities are discussing police involvement in public health-related incidents, this brief contribution describes a year’s worth of public health related calls to police in one city, as evidenced through police administrative records.

To examine a typical period, a pre- COVID-19 year was selected. In 2019, Philadelphia, Pennsylvania, was the 6th largest city in the United States with a population of 1.58 million comprising 34.2% White, 40.1% Black, and 15.2% Hispanic. It had a median household income of just under $46,000, and a quarter of the city lived in poverty.^1^ Among over 15,000 violent crimes and 50,000 property offenses, the city recorded 356 homicides (a rate of 22.5 per 100,000 and 4.5 times higher than the national rate).

## Medical/public health related incidents

The Philadelphia Police Department’s (PPD) computer-aided dispatch (CAD) database contains over 3.3 million entries for 2019, of which just over 1 million (1.07 million) were unique calls for service from the public to which at least one of the city’s 6584 sworn officers^2^ was dispatched. Call classification (also called 'slotting' or 'recoding', Gillooly, 2020) involves interpreting the call information and assigning a relevant organizational category, type or code. As Neusteter, Mapolski, Khogali, and O’Toole (2019: 9) note “A handful of codes is likely insufficient to cover all eventualities”, and PPD has over 130 CAD codes, ranging from assaults to vandalism. Selecting one of these codes reflects the initial ‘best guess’ classification of an event by the dispatcher and does not necessarily indicate the final disposition after police attend the incident.

Table 1 summarizes the 1.07 million dispatched events. Rather than list over 80 CAD codes,^3^ they are grouped here into general categories such as calls to help the *community* manage non-crime problems, active *crime* events or reports of recent crime, *medical*-type activities or calls related to public health, *proactive* work generated by the public to suspicious activity, *quality* of life incidents that may run contrary to the usual and tranquil flow of neighborhood life, and *traffic* activity.^4^ What appear (at least initially at the point of dispatch) to be crime and quality of life incidents comprise nearly two-thirds of dispatched calls from the public.

**Table 1:** General CAD event categories with frequencies and equivalent officer shift activity time

Event type	Frequency	Percent	Officer shifts	Percent
Community	108,158	10.2	11,016	7.2
Crime	425,337	39.9	83,458	54.7
Medical/public health	79,211	7.4	13,097	8.6
Proactive	80,804	7.6	7,525	4.9
Quality	251,990	23.7	20,850	13.7
Traffic	119,944	11.3	16,690	10.9
Total	1,065,444	100	152,636	100

Note that percentages do not sum exactly to 100 due to rounding. A shift was estimated at 7.5 h (8 h minus a 30-min refreshment break)

The number of calls is rarely indictive of how long officers spend on each activity. This can be calculated by taking the time spent on each call (from when the call was dispatched to the officer until the officer closes out the event) and multiplying by the number of patrol officers who attended the incident. While at the initial dispatch level what appear to be medical/public health events comprise 7.4% of calls, the nearly 80,000 calls take up the equivalent of over 13,000 total personnel shifts (7.5 h per shift). This represents 8.6% of all committed time by police to calls from the public (Table 1).

**Fig. 1.**
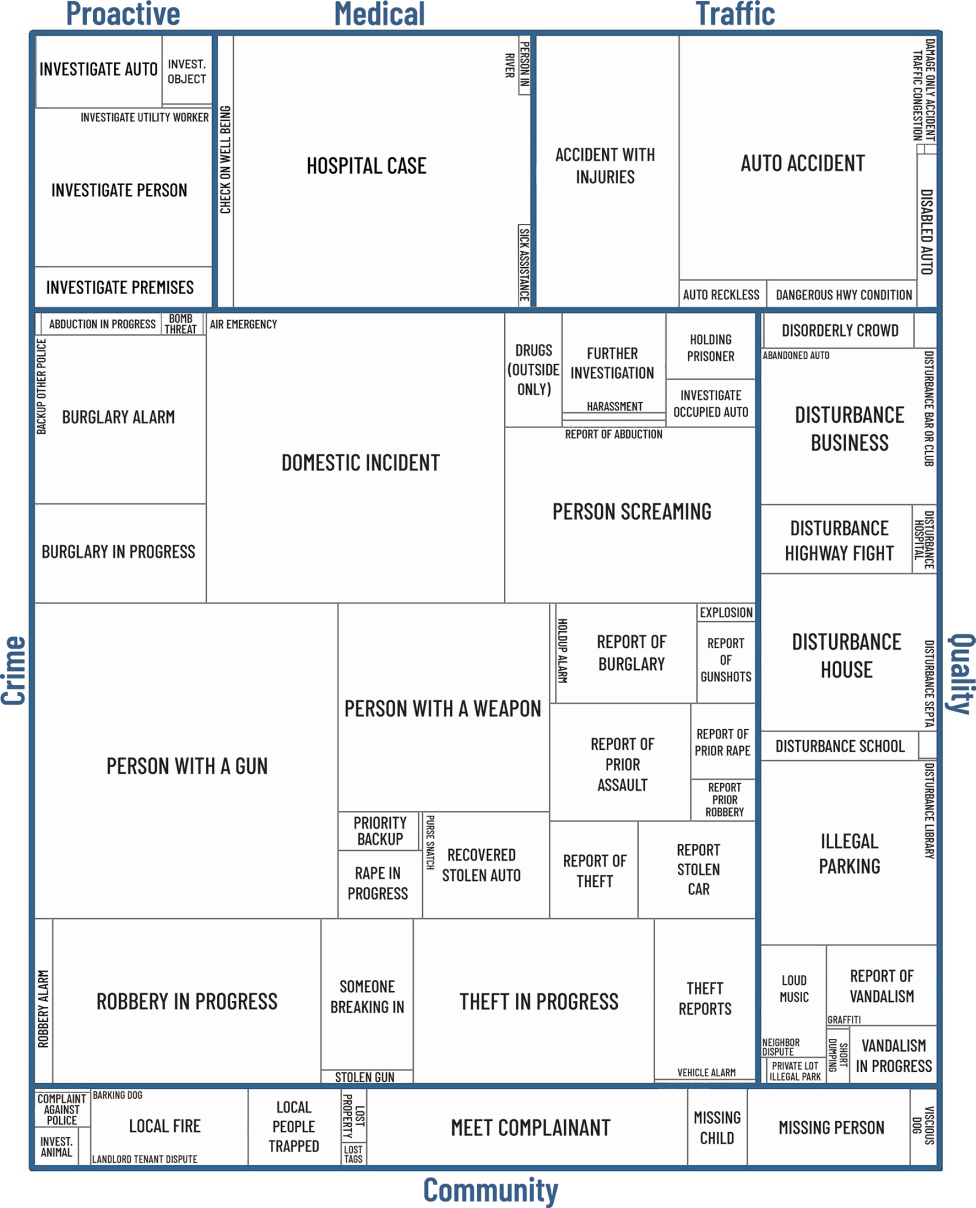
2019 dispatched incidents that originate with the public, weighted by total time officers spend on each call type, Philadelphia, PA. See footnote 4

For more detail, in Fig. 1 box size indicates the time spent on each CAD code type in 2019, based on initial classification assigned by the dispatcher and weighted for the number of officers in attendance. The *crime* category dominates (54.7% of time committed, see Table 1), mainly consisting of domestic incidents, people with weapons, and robberies and thefts in progress. In discussions with CAD supervisors and PPD personnel, four CAD codes were associated with a public health nexus (see footnote 4). A category for reports of people in one of the city’s rivers (144 such incidents in 2019), a ‘*sick assist*’ call for general assistance, a welfare check (*check on well-being*), and a large catch-all category for ‘*hospital case*’. In 2019, Philadelphia did not disaggregate mental health type calls at the CAD entry level (though CAD free-text notes not available to the author might reflect this).

## Spatio-temporal patterns

**Fig. 2.**
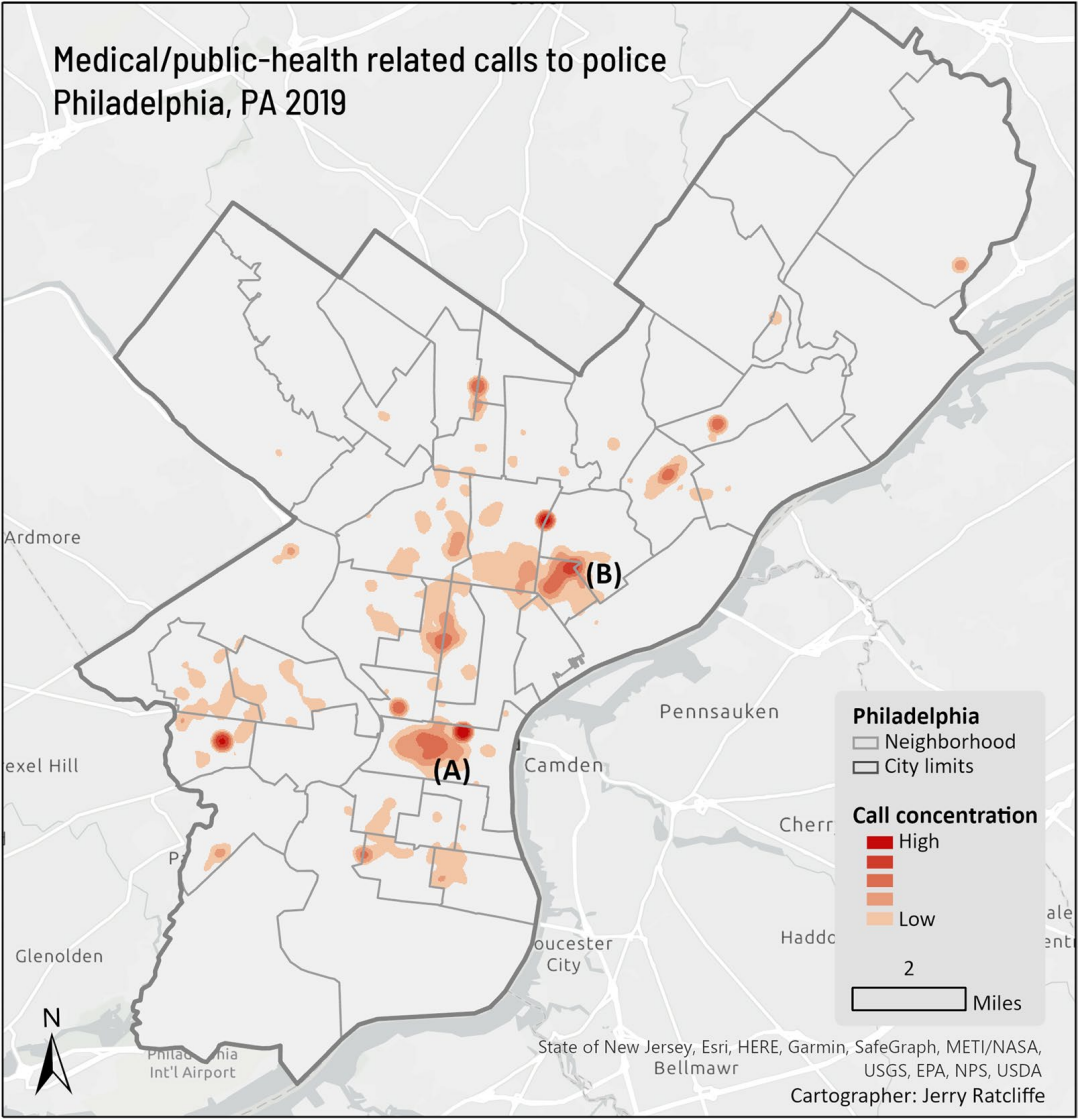
2019 medical/public health-related police call concentrations, Philadelphia, PA

**Fig. 3.**
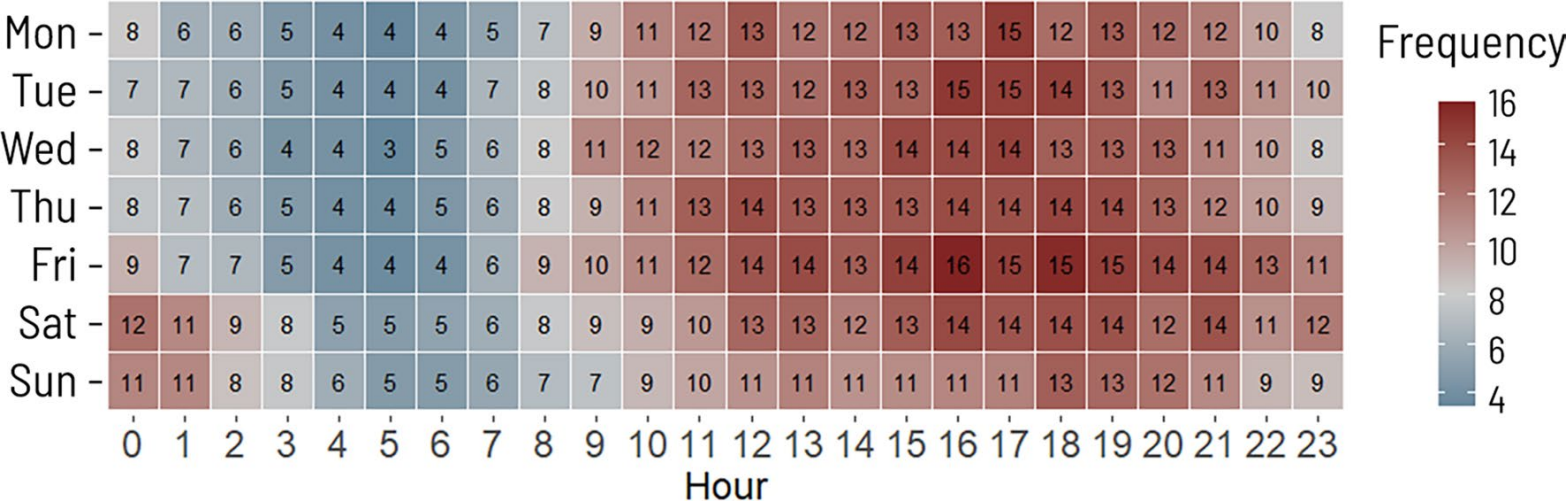
2019 health-related public calls for police service, citywide average per hour, Philadelphia, PA

As with criminal activity, and a characteristic not unique to Philadelphia (Koziarski, in press), health-related policing calls are concentrated in space and time. In Philadelphia, public health calls concentrate in the Center City (A in Fig. 2) and Kensington (B) neighborhoods of the city. Both are focal areas for the homeless populations, with the latter recently referred to as “the largest open-air heroin market on the East Coast of the United States” (Johnson et al., 2020: 3). Health-related policing calls are up to four times more frequent in the afternoon and evening compared to the early hours of the morning (Fig. 3) with on average 15 calls per hour on Friday afternoons and early evening.

## From initial call to final disposition

**Fig. 4:**
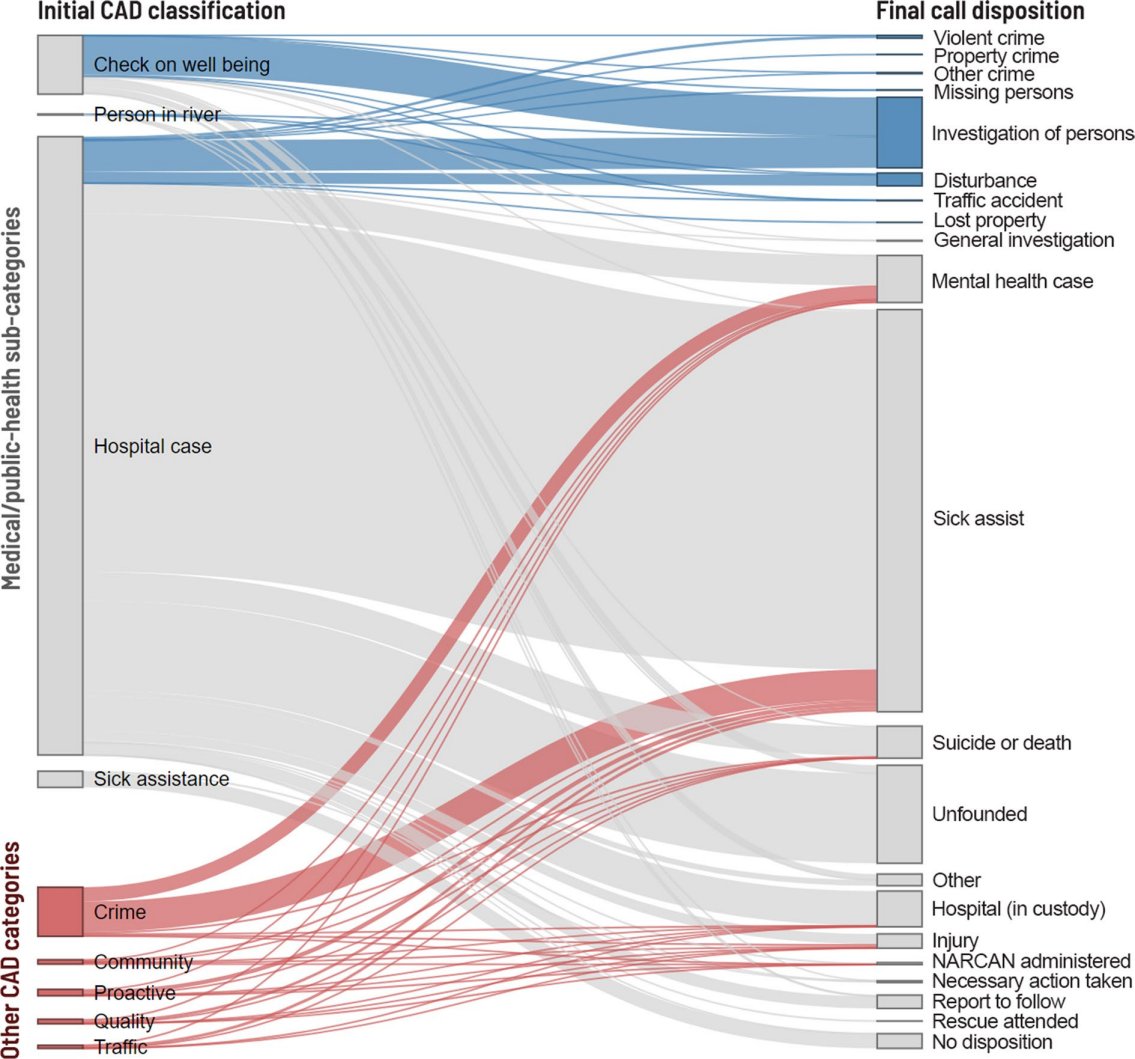
2019 medical or public health-related PPD CAD event classification and disposition, Philadelphia, PA

Given the limitations of the dispatch process, once the officer arrives on scene, the event’s final disposition may be different than suggested by the initial CAD classification (Simpson, in press). This is evident in Fig. 4. The nearly 80% of health-related calls to police that go on to result in a health-related outcome are shown in grey. Blue lines reflect the 11.6% of calls that originate as a health-related CAD event but result in a crime or other policing outcome, and red lines indicate the 8.8% of events that originate as non-health related, but on investigation by police result in a health-type disposition.

## Conclusion

This brief contribution seeks to inform the current discussion around the public health role of the police. In Philadelphia, at least in a relatively normal (i.e. non-COVID-19) year, calls to the police that start or result in some form of medical/public health connection comprise about 8% of the police activity that originates from the public. Many of these incidents reflect policing’s social service role, and could be dealt with by other agencies, though the final call disposition is not easily predicted from the initial CAD classification. Sometimes the only way to determine if a police response is needed is to send a police response.

## Data Availability

The data are not available as they are confidential and contain health-related information.

## Footnotes

1 https://www.census.gov/quickfacts/philadelphiacitypennsylvania.

2 https://ucr.fbi.gov/crime-in-the-u.s/2019/crime-in-the-u.s.-2019/tables/table-1.

3 While PPD has over 130 CAD codes, many do not originate with the public or require dispatching a police officer. These CAD events are excluded from this analysis.

4 These general categories are not used by PPD. They only assign and dispatch the 130 or more CAD codes. The overview categories are used here to illustrate broad classifications of event types and are for descriptive summary purposes only. CAD events were classified after consultation and subsequent refinement from a CAD supervisor and staff in PPD offices for research, planning and personnel, though it is acknowledged that CAD events can be classified in different ways. Police-initiated activity (not reflected here) can also result in medical/health outcomes; however, this contribution focuses on calls that originate when the public call police.

## References

[CR1] Bittner E (1967). The police on skid-row: a study of peace keeping. American Sociological Review.

[CR2] Dijk AV, Crofts N (2017). Law enforcement and public health as an emerging field. Policing and Society.

[CR3] Gillooly JW (2020). How 911 callers and call-takers impact police encounters with the public: The case of the Henry Louis Gates Jr. arrest. Criminology and Public Policy.

[CR4] Goldstein H (1979). Improving policing: A problem-oriented approach. Crime and Delinquency.

[CR5] Johnson NJ, Roman CG, Mendlein AK, Harding C, Francis M, Hendrick L (2020). Exploring the influence of drug trafficking gangs on overdose deaths in the largest narcotics market in the eastern United States. Social Sciences.

[CR6] Koziarski, J. (in press). Examining the spatial concentration of mental health calls for police service in a small city. *Policing: A Journal of Policy and Practice*.

[CR7] Neusteter, S. R., Mapolski, M., Khogali, M., & O’Toole, M. (2019). *The 911 Call Processing System: A Review of the Literature as it Relates to Policing*. New York: Vera Institute of Justice. www.vera.org/publications/911-call-processing-system-review-of-policing-literature.

[CR8] Simpson, R. (in press). Calling the police: Dispatchers as important interpreters and manufacturers of calls for service data. *Policing: A Journal of Policy and Practice*.

[CR9] Wood JD (2020). Private policing and public health: a neglected relationship. Journal of Contemporary Criminal Justice.

[CR10] Wood JD, Watson AC, Barber CWJ (2021). What can we expect of police in the face of deficient mental health systems? Qualitative insights from Chicago police officers. Journal of Psychiatric and Mental Health Nursing.

